# Metabolic networks for nitrogen utilization in ***Prevotella ruminicola*23**

**DOI:** 10.1038/s41598-017-08463-3

**Published:** 2017-08-10

**Authors:** Jong Nam Kim, Celia Méndez–García, Renae R. Geier, Michael Iakiviak, Jongsoo Chang, Isaac Cann, Roderick I. Mackie

**Affiliations:** 1 Department of Animal Sciences, University of Illinois, Urbana, Illinois USA; 2 Department of Agricultural Sciences, Korean National Open University, Seoul, Korea; 3 Carl R. Woese Institute for Genomic Biology, University of Illinois, Urbana, Illinois USA; 4 Department of Microbiology, University of Illinois, Urbana, USA; 5 Department of Beef and Dairy Science, Korea National College of Agriculture and Fisheries, Jeonju, Korea

## Abstract

Nitrogen metabolism in gut systems remains poorly studied in spite of its importance for microbial growth and its implications for the metabolism of the host. *Prevotella* spp. are the most predominant bacteria detected in the rumen, but their presence has also been related to health and disease states in the human gut and oral cavity. To explore the metabolic networks for nitrogen assimilation in this bacterium, changes in gene expression profiles in response to variations in the available nitrogen source and to different concentrations of ammonium were analyzed by microarray and reverse transcription quantitative PCR, and linked with function by further proteomic analysis. The observed patterns of transcript abundances for genes involved in ammonium assimilation differed from the classical “enteric paradigm” for nitrogen utilization. Expression of genes encoding high substrate affinity nitrogen assimilation enzymes (GS-GOGAT system) was similar in growth-limiting and non-limiting nitrogen concentrations in *P. ruminicola* 23, whereas *E. coli* and *Salmonella* spp. responses to excess nitrogen involve only low substrate affinity enzymes. This versatile behavior might be a key feature for ecological success in habitats such as the rumen and human colon where nitrogen is rarely limiting for growth, and might be linked to previously reported *Prevotella* spp. population imbalances relative to other bacterial species in gut systems.

## Introduction

The genus *Prevotella* constitutes one of the most predominant genera in the rumen, with major roles in carbohydrate and nitrogen metabolism^1–3^. The contribution of ruminal *Prevotella* spp. to carbon metabolism in the rumen has been explored extensively^4, 5^, whereas its contributions to nitrogen metabolism have not been studied in detail despite its crucial importance to the growth and performance of the ruminant. *Prevotella ruminicola* strain 23 has been a subject of research since its isolation and characterization by Bryant *et al*. in 1958^4^. The bacterium is non–cellulolytic, but can efficiently degrade hemicellulose and pectin^6^, and could potentially degrade the proteoglycan of the host, as suggested by its genomic content^7, 8^. Regarding nitrogen metabolism, *P. ruminicola* 23 can efficiently utilize both ammonia and peptides (preferentially larger peptides, up to 2,000 Da) as a nitrogen source for growth^8, 9^. For breakdown of oligopeptides, *P. ruminicola* 23 harbors the greatest range and specific activity of dipeptidyl peptidases in comparison to species belonging to other abundant genera in the rumen, such as *Butyrivibrio*, *Ruminococcus*, and *Fibrobacter* ^10, 11^.

A key aspect of the nitrogen cycle in rumen bacteria is that ammonium is the preferred nitrogen source for growth^9^. The enzymes controlling the process are the redox dependent glutamate dehydrogenase (GDH, encoded by *gdhA*), which catalyzes the direct incorporation of ammonium into α–ketoglutarate (α–KG) and the ATP dependent glutamine synthetase (GS, encoded by *glnA* and *glnN* genes), coupled with the glutamate synthase (or glutamine oxoglutarate aminotransferase, GOGAT, a 2 subunit enzyme encoded by *gltB* and *gltD*), which allows the interconversion of glutamate and glutamine by operating cyclically in the GS–GOGAT pathway^12^. *P. ruminicola* 23 encodes two different GDH enzymes (NAD– and NADP–dependent GDHs)^13^ and three distinct GS enzymes (GSI, encoded by *glnA*; GSIII–1, *glnN*−1 gene; and GSIII–2, *glnN*−2 gene)^11^. Ammonium uptake for biosynthetic purposes is mediated by the membrane bound AmtB transporter (encoded by *amtB*). Regulation of the process involves the ubiquitous nitrogen regulatory protein P_II_, encoded by *glnK*. The activity of the implicated enzymes is controlled at both the genetic and enzymatic levels, depending on the extracellular nitrogen concentration^12^.

A few studies have shown that *Prevotella* spp. regulate ammonium assimilation enzymes in response to different nitrogen sources and concentrations, e.g., increased GDH and GS activities have been reported after growth under low nitrogen conditions for *P. ruminicola* 23 and *P. bryantii* B_1_4^13, 14^, and after growth on peptides in *P. ruminicola* 23 and *P. brevis* GA 33^13^. Although these studies have shown regulatory differences occurring between nitrogen sources and concentrations, the function of individual enzymes and the regulation of their coding genes remained to be characterized.

In the current work, we investigate whole genome transcriptional responses to environmental changes in the available nitrogen source and ammonium concentrations in *P. ruminicola* 23 in defined medium and culture conditions, as well as proteome changes and the enzymatic activity of central enzymes in ammonium assimilation. Our study reveals key biochemical aspects in the responses of strain 23 to environmental nitrogen that contribute to our understanding on the ecological success of the genus *Prevotella* under prevailing nitrogen environmental conditions.

## Results

### Growth kinetics of *Prevotella ruminicola* 23 on different nitrogen sources and concentrations

**Figure 1:**
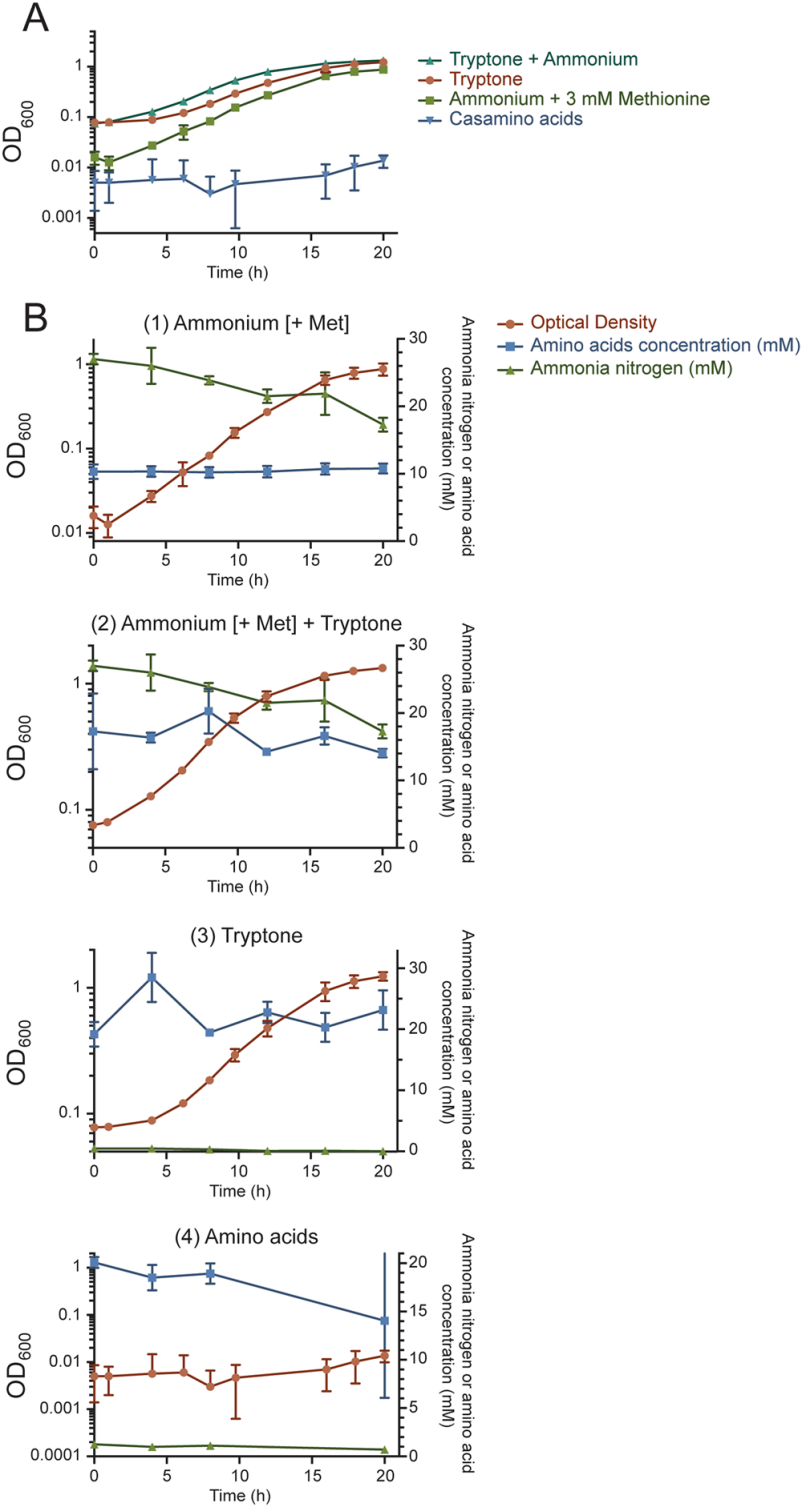
(**A**) *Prevotella ruminicola* 23 can utilize ammonium sulfate supplemented with 3 mM methionine (green line), peptides (tryptone, orange), and a mixture of both (light blue) for growth. The bacterium displayed no growth using amino acids as the sole nitrogen source (dark blue). Growth was measured by determining average optical densities at 600 nm (OD_600_) on triplicate tubes. Error bars represent standard deviations. (**B**) Combined substrate depletion and growth curves indicated that growth on ammonium sulfate + Met corresponded to a decrease in the concentration of ammonia nitrogen (1); growth on tryptone + ammonium sulfate + Met showed a decrease in ammonium nitrogen and amino acids, the latter indicating depletion of peptides (2); growth on tryptone was supported by a slight decrease in ammonia nitrogen and amino acids (3); and lack of growth on casamino acids was linked with insignificant changes in their concentration during growth (4).

Utilization of nitrogen resources in *Prevotella ruminicola* 23 was evaluated by growth in batch culture on different nitrogen sources, including peptides (tryptone), ammonium + Met (ammonium sulfate supplemented with methionine), or amino acids (casamino acids) in a nitrogen-minimal medium, and also in chemostat culture (Supplementary Fig. S2) on different ammonium (ammonium sulfate + Met) concentrations (excess and growth–limiting). The basal medium included 0.05% cysteine as a reducing agent due to early reports that strain 23 required cysteine for initiation of growth and the existence of erratic and poor growth using Na_2_S as reducing agent^9^. The bacterium utilized ammonium sulfate supplemented with 3 mM methionine and peptides (tryptone) as nitrogen sources for rapid growth (0.30 h^−1^ growth rate on ammonium sulfate + Met, and 0.24 h^−1^ on peptides) (Table 1), reaching similar OD values (~1) after 20 h of growth (Fig. 1A). Growth was not observed (ΔOD < 0.01) in the defined medium on ammonium sulfate without Met supplementation (Table 1). Strain 23 displayed no growth using amino acids as the sole nitrogen source (Fig. 1A), even in the presence of cysteine as reducing agent. Analysis of substrate depletion in batch culture (Fig. 1B) showed a decrease in the concentration of ammonium sulfate, while the amino acid concentration remained stable, indicating that ammonium sulfate is the main nitrogen source under this condition (Fig. 1B(1)). Growth on ammonium sulfate and tryptone also corresponded with substrate depletion (Fig. 1B(2)). Minor variations in the concentration of peptides correlate with the observed rapid growth on tryptone (Fig. 1B(3)). A slight decrease in the concentration of amino acids was also consistent with the observed absence of measurable growth on casamino acids (Fig. 1B(4)).

**Table 1:** Effect of different nitrogen sources on growth of *P. ruminicola* strain 23.

Nitrogen source	Specific growth rate (h^−1^)^*^	Maximum cell density (OD_600_)^**¥^
Ammonium + 3 mM Met	0.30	0.95 ± 0.15
Ammonium	0	<0.01
Peptides	0.24	1.31 ± 0.05
Peptides + Ammonium	0.25	1.40 ± 0.03
Amino acids	0.001	0.02 ± 0.0

(*) Number of generations per unit time during exponential growth.

(**) Maximum optical density values after 38 hours culture.

(¥) Average values from three technical replicates.

**Figure 2:**
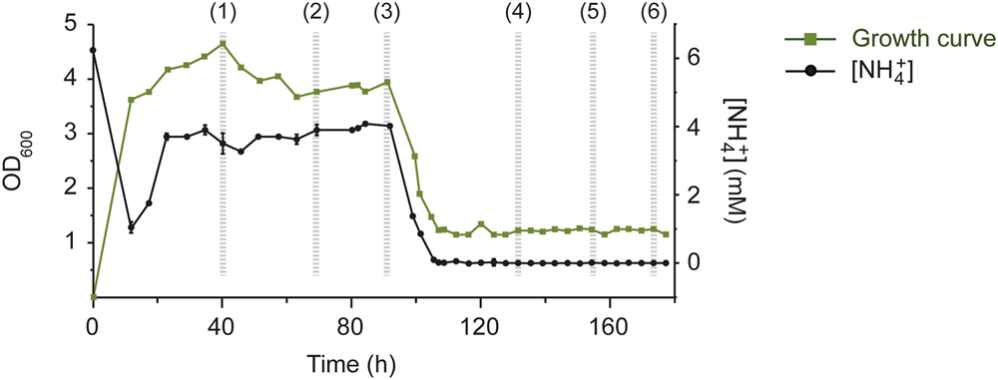
Growth of *P. ruminicola* 23 in continuous culture shifting from excess ammonium (10 mM) to limiting conditions (0.7 mM), and residual ammonium concentration in culture medium. Higher ammonium concentrations yielded higher ODs in comparison with growth–limiting conditions. Growth was measured by determining average optical densities at 600 nm (OD_600_) in triplicate. Error bars represent standard deviations.

In chemostat culture, higher ammonium concentrations yielded higher ODs (OD_600_ 3.90 ± 0.28) in comparison to growth–limiting conditions (OD_600_ 1.19 ± 0.06) (Fig. 2). The residual ammonia concentration was 3.66 ± 0.21 mM in the effluent following growth under excess concentrations of ammonia, indicating that the nitrogen source was non limiting. Conversely, residual ammonium was below detectable levels (<0.025 mM) during growth under ammonia limiting conditions (Fig. 2).

### Genes involved in nitrogen metabolism in *P. ruminicola* 23

**Figure 3:**
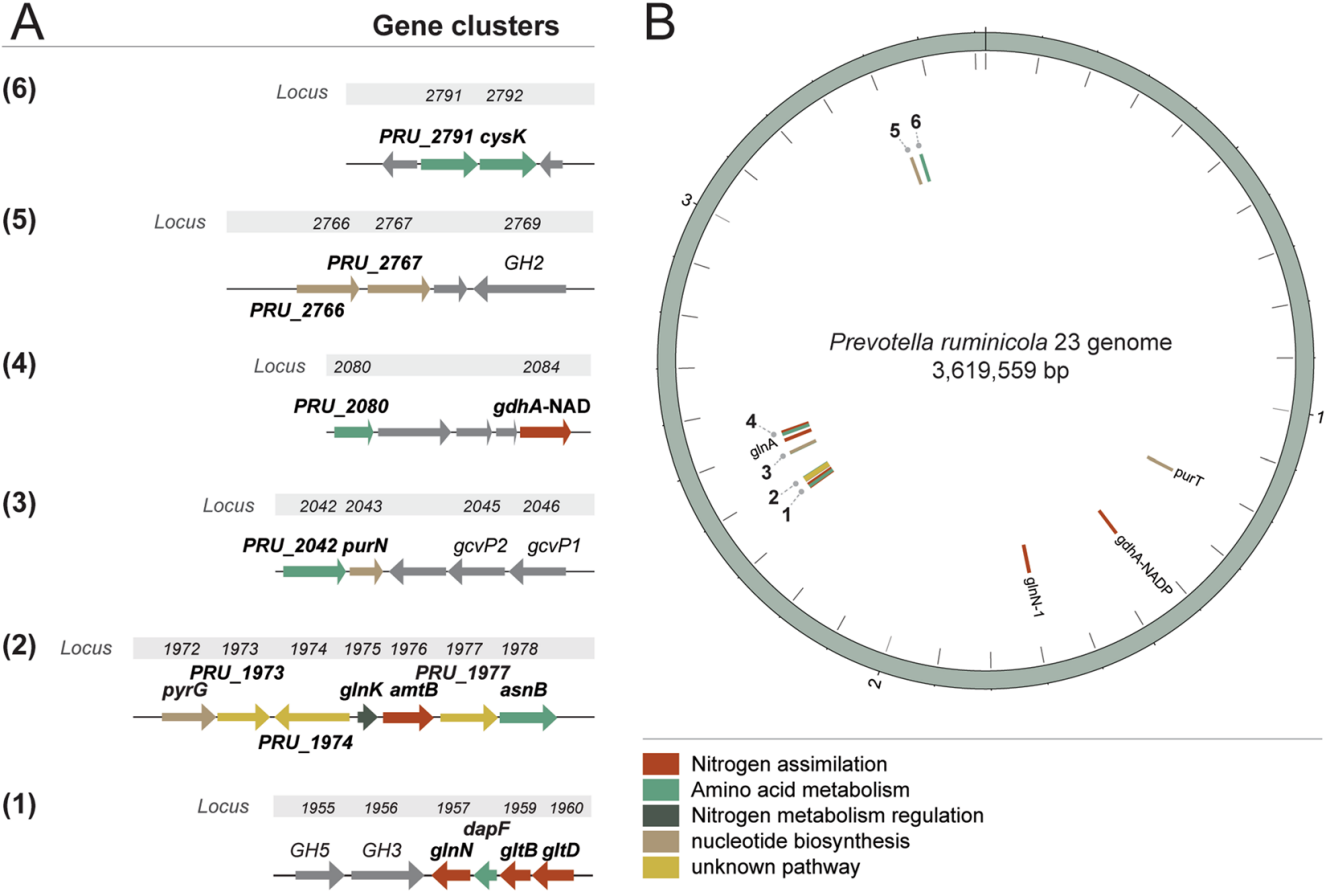
Gene clusters (1 to 6) for nitrogen uptake and assimilation in the genome of *Prevotella ruminicola* 23 (**A**), and genome map with corresponding numbered locations and gene coordinates (**B**). Color code represents functional categories for genes relevant to nitrogen metabolism in *Prevotella ruminicola* 23.

The genomic context of nitrogen metabolism–related genes in strain 23 was characterized by the presence of two main clusters for ammonium uptake, assimilation, and its regulation. ORFs for the nitrogen regulatory protein P_II_, ammonium transporter, a protein of unknown function (locus tag PRU_1977), and asparagine synthase B configure one cluster downstream of the genes encoding enzymes involved in ammonium assimilation (GSIII–2, *glnN*-2 gene, and GOGAT, *gltBD*), which cluster together with the diaminopimelate epimerase gene (*dapF*), implicated in lysine biosynthesis (Fig. 3A(1,2)). The coding sequences for the GSI (*glnA*) and GSIII-1 (*glnN*-1) are found at discrete regions within the genome (Fig. 3B). Genes involved in nucleotide metabolism (*purN* and *purT*, encoding two different phosphoribosylglycinamide formyltransferases, and *pyrG*, encoding a CTP synthase) are found scattered across the genome (Fig. 3A(2,3,5),B). The gene encoding the diaminopimelate dehydrogenase (PRU_2042), also involved in lysine biosynthesis, and the phosphoribosylglycinamide formyltransferase (*purN*), involved in purine biosynthesis, are located together (Fig. 3A(3)). Vitamin metabolism genes [phosphomethylpyrimidine kinase (PRU_2766) and pyridoxine biosynthesis protein (PRU_2767) genes], or with unknown pathway assignment [aminotransferase (PRU_1974), glutamine amidotransferase (PRU_1973)], are also found in defined clusters in the genome of strain 23 (Fig. 3A(2,5)). Genes involved in cysteine metabolism are found clustered together (O–acetylhomoserine aminocarboxypropyltransferase, PRU_2791, and cysteine K, *cysK*, genes) (Fig. 3A(6)). An additional coding sequence for a O–acetylhomoserine aminocarboxypropyltransferase appears upstream of the NAD-dependent glutamate dehydrogenase (*gdhA*-NAD gene) (Fig. 3A(4)), which is located separately from the NADP-dependent enzyme (Fig. 3B).

### Differentially expressed genes in response to ammonium concentration

**Figure 4:**
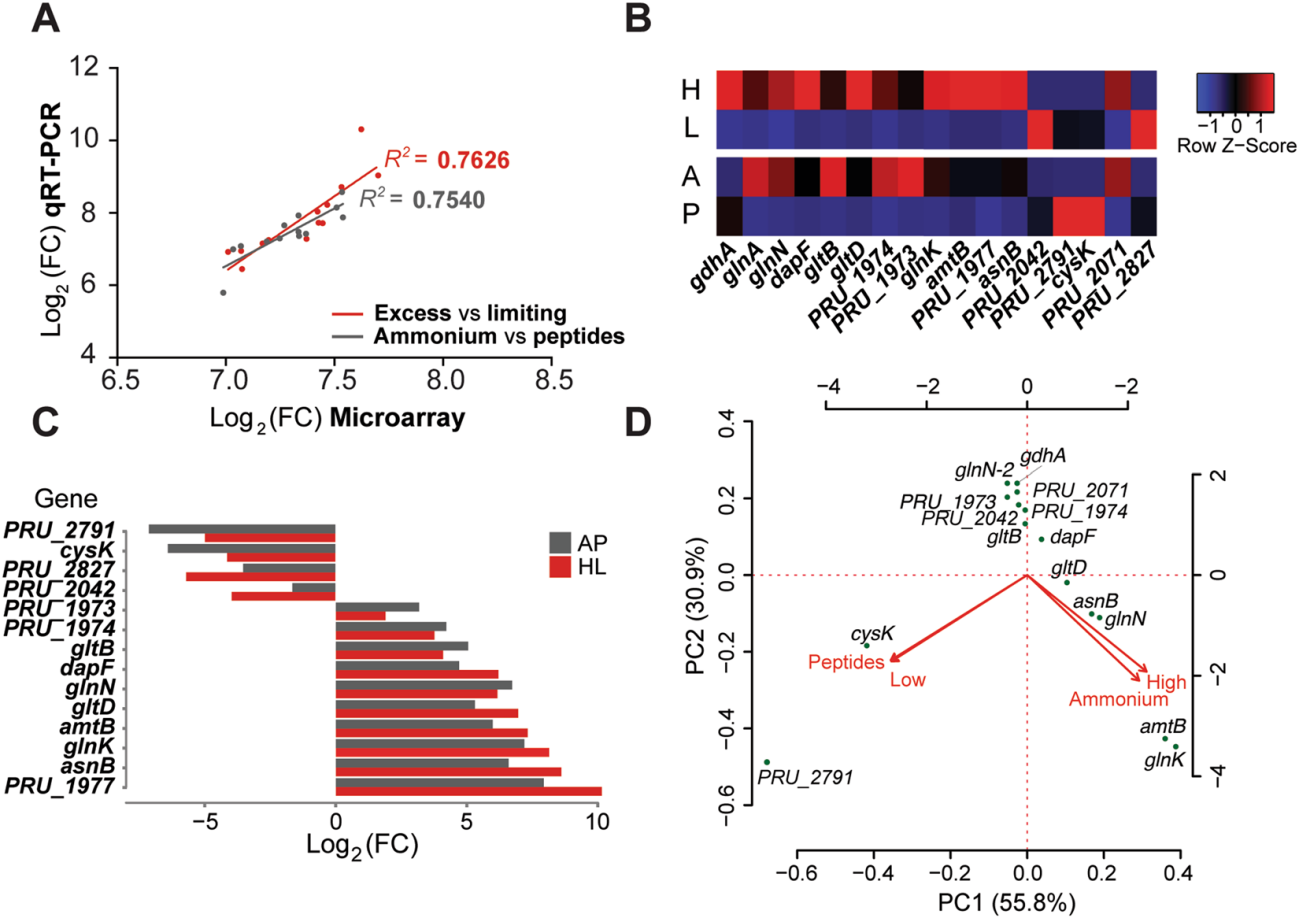
(**A**) Log transformation of the fold change in transcript abundances obtained through microarray and qRT-PCR indicates consistency in the results obtained through both techniques (**B**) Heat map displaying normalized changes in transcript abundances for a subset of genes implicated in nitrogen metabolism. Highest obtained values under non-limiting nitrogen conditions corresponded to genes related to ammonium transport (*amtB*, ammonium transporter gene; PRU_1977, hypothetical transporter), ammonium assimilation and its regulation (PRU_2048, NAD-dependent glutamate dehydrogenase; PRU_2071, *gdhA*, NADP-dependent glutamate dehydrogenase; *glnA*, glutamine synthetase type I; *glnN*-1 glutamate synthetase type III-1; *glnN*-2 glutamate synthetase type III-2; *gltB*, glutamate synthase, large subunit; *gltD*, glutamate synthase, small subunit; *glnK*, nitrogen regulatory protein P_II_), amino acid and protein biosynthesis (*dapF*, diaminopimelate epimerase; *asnB*, asparagine synthase; PRU_1974, aminotransferase, homolog; PRU_1973, glutamine amidotransferase). Highest values under growth on peptides corresponded to genes involved in protein biosynthesis (PRU_2971, O-acetylhomoserine aminocarboxypropyltransferase; *cysK*, cysteine synthase; PRU_2042, diaminopimelate dehydrogenase), or had unclear roles in nitrogen metabolism (PRU_2827, outer membrane receptor RagA) (H, growth on excess ammonium; L, growth on limiting ammonium concentrations; A, growth on ammonium; P, growth on peptides). (**C**) Comparison of log transformed fold changes in transcript abundances obtained by qRT-PCR in the assayed growth conditions (AP, Ammonium vs Peptides, HL, excess (H) vs growth-limiting (L) ammonium concentrations). Gene symbols and observed trends as in (**B**). (**D**) Principal component analysis integrating fold change in transcript abundances obtained through microarray and qRT-PCR on *P. ruminicola* 23 grown in different nitrogen sources (ammonium or peptides) or ammonium concentrations (excess [high] or growth-limiting [low]). Arrows point in the direction of the maximum correlation. Gene symbols as in (**B**) and (**D**).

*P. ruminicola* 23 genes were differentially expressed during growth under different concentrations of ammonium (excess or limiting for growth). Microarray and qPCR data showed that fold changes in transcript abundances between conditions were consistent (Fig. 4A). When grown on excess concentrations of ammonium, 65 genes of strain 23 displayed a change 4–fold higher (30 genes) or lower (35) in their transcript abundances (Supplementary Table S2). Among them, 18 (*amtB*, *glnK*, *asnB*, *gdhA*, *glnA*, *glnN*, *dapF*, *gltB*, *gltD*, *purN*, *purT*, *pyrG*, *cysK*, PRU_2042, PRU_2080, PRU_2766, PRU_2767, PRU_2791) are known to be involved in nitrogen metabolism. Reverse transcription quantitative PCR and microarray data showed consistently that genes involved in ammonium transport (*amtB*), ammonium assimilation (*gdhA*, *glnN*, *gltBD*) and its regulation (*glnK*), and amino acid and protein biosynthesis (*asnB*), were strongly induced under limiting conditions of ammonium (Fig. 4B,C). The highest significant (p-value < 0.01) fold changes in transcript abundances after comparison between conditions corresponded to the following clustered genes: the ammonium transporter gene (*amtB*, 69.1 fold change), the gene encoding a conserved hypothetical, presumptive transmembrane protein (Supplementary Fig. S3) (PRU_1977, 58.3 fold change), the nitrogen regulatory protein P_II_ gene (*glnK*, 46.2 fold change), and the asparagine synthase B gene (*asnB*, 38 fold change) (Supplementary Table S2; Fig. 4B,C) (fold changes correspond to normalized microarray signal intensities). Transcript abundances of genes encoding enzymes implicated in ammonium assimilation were 2.2 fold higher for the NADP–specific GDH gene (*gdhA*), 35.4 fold higher for the GSIII–2 gene (*glnN*), and 32.7 fold/26.6 fold higher for the small/large GOGAT subunits (*gltB and gltD*), respectively.

Genes involved in protein fate, energy metabolism, DNA metabolism, signal transduction, regulation, and biosynthesis of cofactors, prosthetic groups and carriers, showed higher transcript abundance levels when *P. ruminicola* 23 was grown on limiting concentrations of ammonium (Supplementary Table S2). Specifically, genes encoding carbohydrate (PTS galactitol family and a fucose:hydrogen symporter) and amino acid/peptide transporters (peptide ABC transporter, polyamine ABC transporter), genes implicated in the degradation of proteins/peptides/glycopeptides (peptidases), as well as an anaerobic C4–dicarboxylate (succinate, fumarate, malate) membrane transporter (protein DcuB), and cysteine metabolism–related genes, were slightly induced in these conditions (Supplementary Table S2). Genes encoding the cysteine synthase K and the O–acetylhomoserine aminocarboxypropyltransferase/cysteine synthase (*cysK* and *dapF*, respectively), involved in amino acid biosynthesis, showed more than 10 fold change lower transcript abundances in ammonium–limiting conditions (Supplementary Table S2). Interestingly, 10 and 16 hypothetical proteins, without predictive roles based on sequence and predicted structure, showed respectively higher or lower transcript abundances after growth in chemostat switching from high to low ammonium concentrations.

### Differentially expressed genes in response to growth on peptides or ammonium

*P. ruminicola* 23 showed differential gene expression patterns during growth on different nitrogen sources. A total of 29 genes displayed more than 4-fold change in their transcript abundances during growth on ammonium or peptides. The genes induced by ammonium encoded enzymes implicated in amino acid biosynthesis (mainly aspartate, lysine, and tryptophan) and transport (ABC–type), ammonium assimilation (GSIII–2, GOGAT), ammonium transport, cell envelope structure and function, energy metabolism, molecular transport and associated binding proteins, or had regulatory functions. The highest detected fold changes in the transcript abundances corresponded to the clustered genes encoding the ammonium transporter (47-fold change, the highest detected), the nitrogen regulatory protein P_II_ (43.3 fold change), the conserved presumptive transmembrane protein (46.5 fold change), and the asparagine synthase B (15.3 fold higher) genes (Fig. 4B,C; Supplementary Table S3). A number of hypothetical proteins (n = 6) showed higher (2) or lower (4) transcript abundances after growth on ammonium or peptides.

### Transcript abundance levels in response to environmental nitrogen

Integration of microarray and qRT–PCR data from both experiments into PCA analysis showed that the expression of the genes encoding proteins involved in ammonium uptake (AmtB), assimilation (GDH, GSIII–2, GOGAT), regulation of ammonium assimilation (nitrogen regulatory protein P_II_), and amino acid synthesis (asparagine and lysine–related, mainly), strongly correlated with growth under non–limiting concentrations of ammonium. Growth on peptides induced expression of the cysteine synthase A, involved in biosynthesis of cysteine (Fig. 4D).

### Evaluation of the activity of enzymes implicated in ammonium assimilation

Enzyme activity and protein localization were analyzed for GDH, GS and GOGAT from cytoplasmic and membrane protein fractions obtained from cells grown under different sources of nitrogen or ammonium concentrations. A higher NADP–GDH, GS, and NADP–GOGAT activity was detected in the cytoplasmic fraction when *P. ruminicola* was grown in non–limiting concentrations of ammonium (Table 2), a fact that correlated with the observed higher transcript abundances of *gdhA*, GSIII–2 gene *glnN*-2, and *gltBD* (Supplementary Tables S2 and S3). None of these enzymes displayed higher activity when growing on peptides. Nevertheless, very low NAD–GDH and NAD–GOGAT activities were detected during growth on both ammonium and peptides.

**Table 2:** Effect of growth on different concentrations of ammonia or peptides in enzyme activities involved in ammonia assimilation in *P. ruminicola* strain 23.

Localization of protein	Ammonium concentration	GDH		GS	GOGAT	
		nmol/mg/min^¥^		nmolP_i_/µg/min^¥^	nmol/mg/min^¥^	
		NADH	NADPH	Biosynthetic	NADH	NADPH
Cytoplasm	Excess	1.1 ± 2.4	24.3 ± 7.3	19.4 ± 2.0	3.5 ± 4.3	14.0 ± 4.9
	Limiting	1.2 ± 2.6	4.6 ± 3.8	8.8 ± 2.6	2.0 ± 2.9	2.4 ± 3.2
Membrane	Excess	0.6 ± 1.4	NM^*^	1.2 ± 1.9	3.4 ± 4.2	1.7 ± 2.2
	Limiting	0.9 ± 2.0	1.2 ± 0.3	0.9 ± 1.3	5.0 ± 5.5	1.9 ± 3.2
	**Nitrogen source**			nmolP_i_/mg/min		
	Ammonia	<0.1	<0.1	8.1 ± 1. 3	<0.1	<0.1
	Peptides	<0.1	<0.1	3.2 ± 2.0	<0.1	<0.1

(*) NM, no activity was detected

(¥) Average values from three technical replicates per condition.

### Proteomic analysis

Ten protein spots obtained through 2D–DIGE (2 Dimensional Difference In-Gel Electrophoresis) analysis from the cytoplasmic fraction in *P. ruminicola* 23 grown on either ammonium or peptides were selected based on intensity for identification purposes (Supplementary Fig. S4). Seven spots (IDs 104, 108, 111, 112, 212, 239, 462) were recovered when the organism was grown on ammonium, while 3 spots (IDs 456, 595 and 598) were isolated from *P. ruminicola* 23 grown on peptides. Analysis through LC–MS/MS was successful for 4 out of 10 proteins (Supplementary Table S4), identified GSIII–2 and GOGAT when growth was on ammonium, and cysteine synthase A and O–acetylhomoserine aminocarboxypropyltransferase/cysteine synthase protein when growing on peptides.

## Discussion

Previous studies had shown that *P. ruminicola* can efficiently utilize peptides and ammonium as nitrogen sources for growth, but not amino acids^8, 9^. Consistent with this, we showed that *P. ruminicola* was unable to sustain growth when supplied with amino acids as the sole nitrogen source. *P. ruminicola* strain 23 showed shifts in its overall transcriptional profiles when growth occurred on ammonium or peptides, e.g. ammonium assimilation pathways were not induced when the bacterium was grown on peptides (Fig. 4). Our results suggest that the bacterium utilizes peptides directly for protein synthesis, but substrate depletion analyses revealed that small changes in the substrate concentration could allow fast growth to high OD values (Fig. 1B(3)). Ammonium nitrogen can be detected during growth on tryptone; therefore, both ammonium and peptides could have supported growth to high OD (Fig. 1B(3)). Substrate depletion analysis after growth on ammonium sulfate and tryptone showed a decrease in both nitrogen sources, indicating that peptides can be used for growth (Fig. 1B(2)). Interestingly, growth on ammonium sulfate was not observed in the absence of supplementation with 3 mM methionine (ΔOD < 0.01, Table 1). Methionine is stimulatory, and might be essential for growth of *P. ruminicola* 23, as previously reported by Pittman and Bryant (1964)^9^, although this is likely not as a N source but as a methyl or methanethiol (CH_3_S) donor in other biosynthetic reactions. Nili and Brooker (1995)^15^ observed that *P. brevis* strain GA33 could not utilize NH_4_Cl as sole nitrogen source, but was able to grow when the medium was supplemented with any amino acid other than methionine, which suggested that the bacterium may generate L–glutamate by transamination between α–ketoglutarate and other amino acids instead of obtaining it through *de novo* synthesis from ammonium and α–ketoglutarate.

### Transcriptional responses of *P. ruminicola* 23 grown on non-limiting ammonium conditions

The presented results collectively show that *P. ruminicola* 23 responds to changes in environmental nitrogen differently from enteric species of Proteobacteria, such as *E. coli* or *Salmonella* spp.^16^. These responses reflect differential transcriptional regulation of genes involved in nitrogen metabolism and variations in related enzyme activities. Transcriptional responses of strain 23 to environmental nitrogen revealed the existence of a similar behavior to enteric bacteria in non–limiting concentrations of ammonium. Previous studies had shown that *P. ruminicola* possesses both NADP– (anabolic) and NAD–dependent (catabolic) GDH activities^13^. Correspondingly, higher transcript abundances of *gdhA* and elevated NADP–GDH activity were detected under excess concentrations of ammonium. The GS–GOGAT pathway constitutes a major ammonium assimilation pathway for enteric bacteria grown under ammonium–limiting conditions^16, 17^. Nevertheless, transcription of GS–GOGAT genes was highly induced in non–limiting concentrations of ammonium in *Prevotella ruminicola* 23 (Fig. 4). In contrast to the enteric paradigm, our results demonstrate that *P. ruminicola* 23 utilizes the high substrate affinity GS–GOGAT enzymatic system to grow in non–limiting ammonium conditions, a pattern that has been also observed in *Ruminococcus albus* 8^18^. To our knowledge, this is the first description of an organism that uses both GDH and GS–GOGAT pathways for ammonium assimilation when grown under non–limiting concentrations of ammonium. Even though the GS–GOGAT system requires ATP, and its down–regulation would prevent energy waste when ammonium assimilation relies on GDH, the observed behavior could reflect simultaneous utilization of GDH and GS–GOGAT pathways to maintain the glutamate pool for the biosynthesis of amino acids. This may represent an evolutionary adaptation of strain 23 to rumen conditions, where nitrogen concentrations are rarely growth limiting (ammonium concentration typically ranges from 4 to 70 mM)^19^. This strategy would enable the organism to maintain an active growth in the natural environment, outcompeting other microbes in the utilization of ammonium as a nitrogen source, consistently agreeing with the observation that *Prevotella* constitutes the most abundant reported bacterial genus in the rumen^20^.

**Figure 5:**
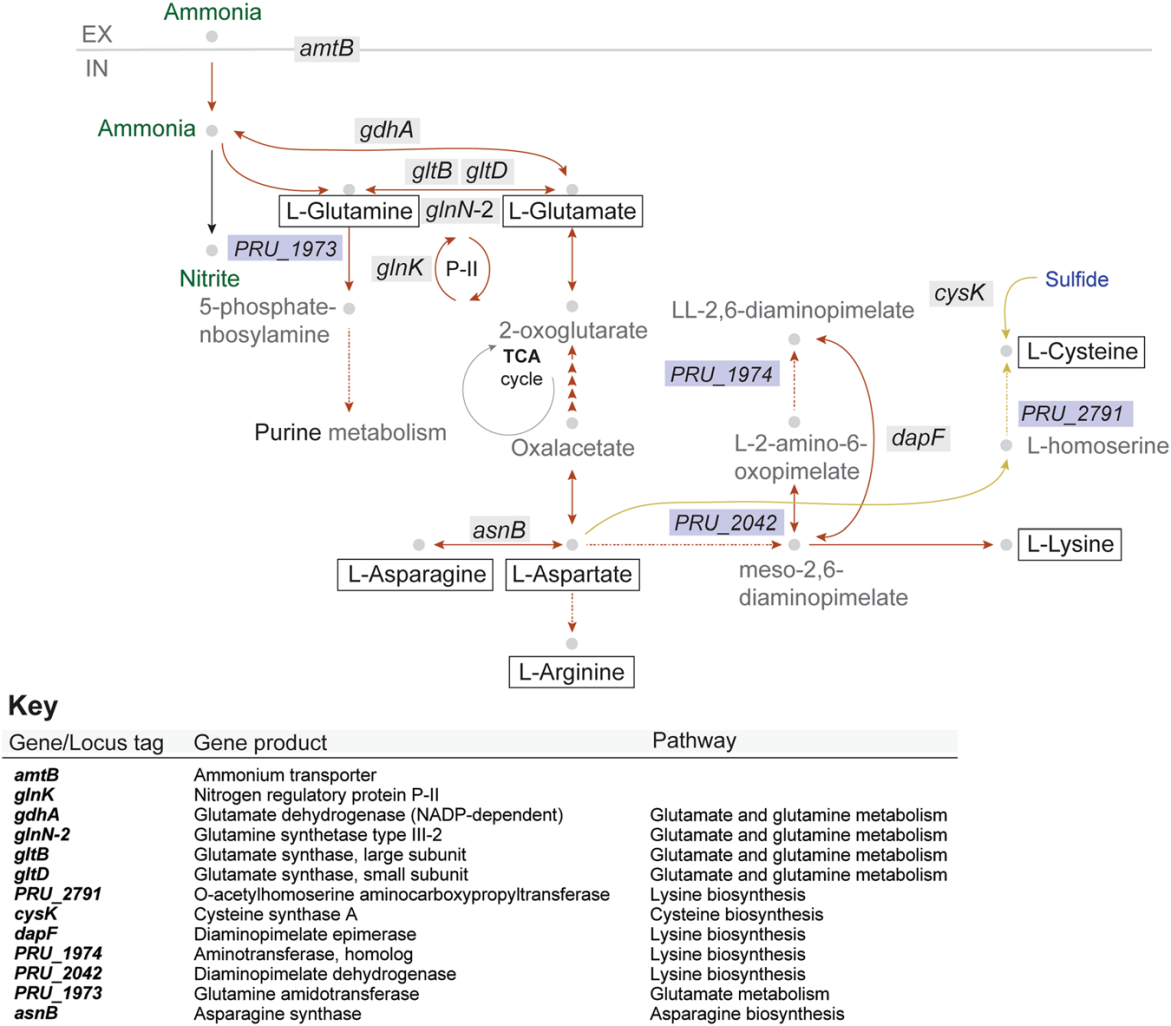
Metabolic networks for nitrogen utilization in *Prevotella ruminicola* 23. The bacterium can utilize both ammonium and peptides for growth through activation of different biochemical pathways. In contrast to *E. coli* and *Salmonella* spp., growth on non-limiting ammonium conditions is maximized by both GDH and GS/GOGAT-dependent ammonium assimilation. Growth on peptides might rely on extracellular hydrolysis and transport of resulting amino acids, or intracellular deamination of imported oligopeptides. High induction of genes involved in cysteine synthesis could indicate generation of labile amino acids in the cell. Chemical/biochemical species are represented by grey circles. Nitrogen-containing species are represented in green, and sulfur-containing species are depicted in blue. EX and IN stand for extracellular and intracellular cell locations, respectively. Dashed arrows are used for clarity in order to represent that many steps are implicated in the generation of the displayed species. Red arrows represent pathways induced on ammonium and yellow arrows represent pathways induced on peptides.

Consistently, protein identification and enzyme activity analyses showed that the main proteins implicated in ammonium assimilation were those displaying most dramatic changes at the gene expression level (Fig. 4; Supplementary Table S4). The collective genomic and proteomic results strongly provide evidence that GSIII–2 is the main enzyme implicated in ammonium assimilation when the nitrogen is non–limiting, in agreement with the observation that the protein plays an important role in the acquisition and metabolism of ammonium under these conditions^11^. Transcript abundances for the GOGAT genes were also higher than 22 fold on ammonium. Therefore, the GSIII-2–GOGAT coupling of enzymes is likely to play a major role in ammonium assimilation and ammonium recycling in *P. ruminicola* 23 (Fig. 5).

The enzyme diaminopimelate epimerase, encoded by *dapF*, catalyzes the production of meso–diaminopimelic acid (Fig. 5), which is both an intermediate in the biosynthesis of lysine and a major component of the cell wall of Gram–negative cell wall type bacteria^21^. The fact that *dapF* displayed one of the highest fold changes in transcript abundances in non–limiting ammonium concentrations appears to directly correlate with cell wall biosynthesis during bacterial growth, and might therefore be indicative of the presence of a successful anabolic strategy for which nitrogen assimilation is key.

Our results showed an increased transcript abundance of the ammonium transporter coding gene (*amtB*) during growth in non–limiting concentrations of ammonium, which suggests the ability of *P. ruminicola* 23 to detect and respond to fluctuations in the environmental ammonium concentration. However, detected transcript abundance levels revealed that, when concentrations of ammonium became limiting, the bacterium induced peptide and polyamine ABC transporters, suggesting its capability to scavenge more complex and/or alternative available nitrogen sources.

Transcript abundances for the nitrogen regulatory protein P_II_ gene, *glnK*, were also higher on non–limiting ammonium. The most recent mechanistic insight into the signaling role of the P_II_ protein suggests that a post translational change driven by changes in its observed ATPase activity under fluctuating nitrogen levels sensed by intracellular α–KG would facilitate or inhibit ammonium uptake through the ammonium transporter channel^22^. The *glnK* gene can be located upstream and downstream of *amtB*. This genetic linkage is highly conserved, and both resulting proteins are functionally related^23^.

The presence of the conserved putative transmembrane protein gene PRU_1977 (ORF2056, Supplementary Table S3) only in *P. ruminicola* 23, and also being the gene displaying the second highest transcript abundance under non–limiting ammonium conditions, raises the question of its implication in the nitrogen metabolism of the bacterium. The highest homology found for ORF2056 was 63% with an uncharacterized protein from *Prevotella copri* isolated from human feces. Functional prediction based on structural alignment against the Protein Data Bank database (30-Apr-16 Release) delivered the highest homology (37.1%, E-value 2e-29) with an integral membrane domain of an auto transporter. Jointly, these predictive results point to a possible role of the protein encoded by the PRU_1977 gene in ammonium transport.

Asparagine synthase B (glutamine hydrolyzing) was also induced under non-limiting ammonium conditions. This enzyme catalyzes the ATP–dependent reversible conversion of L–aspartate into L–asparagine, using glutamine as a source of nitrogen (Fig. 5). Generation of L–asparagine might represent a biochemical strategy to capture nitrogen in the amino acid pool due to the reversible nature of the transformation mediated by AsnB. The *P. ruminicola* 23 holds genomic potential to mediate conversion of L–aspartate to oxalacetate, and the latter to α–KG through a number of enzymes from the citrate cycle. Therefore, nitrogen could be diverted from this molecule for biosynthesis of any amino acid other than Asn, as well as amino sugars, and nucleotides. Also, the rapid generation of Gln through Glu provides the nitrogen source for the generation of asparagine from aspartate.

### Transcriptional responses of *P. ruminicola* 23 grown on peptides

We observed an increase in the transcript abundances of more than 29 ribosomal proteins, arranged in five ribosomal gene clusters generally observed in bacteria^24^, during growth on peptides: *rpoBC*, *str*, *S10*, *spc*, and *alpha*. These genes displayed increased transcript abundances (more than 1.9 fold), with the exception of the *str* cluster, whose genes were constitutively expressed (around 1.2-fold change). This observation provides evidence for increased protein synthesis through the rapid availability of intracellular amino acids from peptide uptake by *P. ruminicola* 23. Whereas no induction of peptidases was observed in the transcriptional profiles, all appearing constitutive (<1.5 fold–change, data not shown) when growth was on peptides, previous studies demonstrated strong peptidase activity in *Prevotella ruminicola* strain 23^25, 26^. Interestingly, we could not detect higher levels of transcript abundances of transporters during growth on peptides. In general, peptide transport occurs via a dipeptide permease or oligopeptide permease, depending on the size of peptides being transported into the bacterium^27^. Overall, the transport mechanism for peptides from the environment by *P. ruminicola* 23 is currently unknown. Peptide metabolism of *P. ruminicola* 23 may be initiated after transport of peptides into the cell, subsequently hydrolyzed to amino acids that can be deaminated or utilized for protein biosynthetic activity (Fig. 5). The genes that appear strongly correlated to growth on peptides were *cysK*, encoding the cysteine synthase A, and the O–acetylhomoserine aminocarboxypropyltransferase (PRU_2791) (Fig. 4D), both involved in the biosynthesis of cysteine. Each enzyme was also detected in the proteomic analysis as one of the major gene products during growth on peptides. This observation might be a reflection of the lability of this amino acid due to the reactivity of its sulfhydryl group in comparison to methionine, therefore being synthesized by the bacterium instead of imported from the environment.

In summary, *P. ruminicola* 23 utilizes both NADP–GDH and the GSIII-2–GOGAT pathways for ammonium assimilation when nitrogen is non–limiting for growth. This may reflect an adaptation of *P. ruminicola* 23 to a more stable concentration of ammonium in the rumen relative to other environments (e.g., human gut)^28^ by enhancing glutamate production and maintaining the intracellular glutamate/glutamine pool for amino acid and cell wall biosynthesis. In contrast, under limiting concentrations of ammonium, *P. ruminicola* 23 may utilize basal levels of GDH or GS–GOGAT pathways to assimilate ammonium and synthesize amino acids.

In Westernized human populations, *Prevotella* predominates in the oral cavity, but it has been also detected in the gut. Gut *Prevotella* are common in non-Western humans who consume a plant-rich diet, which fits with its niche as a gut fermentor, but its presence in the human gut has also been linked with inflammatory conditions and population imbalances relative to *Bacteroides* spp.^29^. Understanding the metabolic networks underlying growth under fluctuating nitrogen conditions could provide of key answers to the causalities of such imbalances, and inspire ideas about population control for improving metabolism or reducing risk of disease.

In the context of evolution, species evolve to maximize energy intake^30^. Ruminants have developed a higher efficient digestive strategy as a result of their symbiotic relationship with rumen microbes. The rarely limiting ammonium conditions in the rumen are representative of an environment in which selection of microbes able to maximize nitrogen assimilation would be adaptively beneficial^31^. According to the more recent rumen census, *Prevotella* spp. are the most abundant group of microbes detected in ruminants across the globe^32^. Available nitrogen in the rumen is the most important predictor of microbial yield^33^; thus, the described strategy for nitrogen assimilation in *Prevotella ruminicola* 23 would allow optimal growth in the natural environment of the bacterium. Whether the same behavior is expressed in the natural environment and in other *Prevotella* spp. remains to be elucidated. Collectively, our results provide a plausible explanation for the ecological success of *P. ruminicola* in the rumen.

## Methods

A summarized schematic of the experimental procedures is presented in Supplementary Fig. S1. Details about protein extraction, protein 2D gels and mass spectrometry for protein identification, and enzyme activity assays can be found in the supplementary information.

### Growth kinetics of *Prevotella ruminicola* 23 on different nitrogen sources and concentrations

*P. ruminicola* strain 23 was provided by M. A. Cotta, USDA–ARS, Peoria, IL. To explore whole transcriptional responses to different nitrogen sources (peptides, ammonium, or amino acids), cells were anaerobically grown at 37 °C in a defined, nitrogen–minimal medium with 0.5% glucose as the carbon source and 0.05% cysteine hydrochloride as a reducing agent^9, 34^, supplemented with either 0.2% tryptone (peptides), 10 mM (NH_4_)_2_SO_4_ + 3 mM methionine (ammonium + Met), 10 mM (NH_4_)_2_SO_4_ without methionine (ammonium), 0.1% tryptone + 5 mM (NH_4_)_2_SO_4  _+ 3 mM methionine (peptides and ammonium + Met), or 0.2% casamino acids (amino acids). Triplicate bottles per nitrogen source were inoculated with 2.5% inoculum at t–0 h (hours). Absorbance readings were taken every 2–4 h until t–24 h. One milliliter samples were removed every 4 h until stationary phase (t–20 h). Each sample was centrifuged for 15 minutes at 16,000 × *g*. Supernatants were separated from the cell pellet and frozen at −80 °C.

### Analysis of substrate utilization

To evaluate substrate depletion, supernatants were assayed for ammonium and amino acid concentration. For RNA and protein extractions, *P. ruminicola* 23 cultures were harvested at mid-log phase by centrifugation at 10,000 × *g* for 5 min. The cell pellet from the centrifugation and supernatant were stored in RNAprotect bacteria reagent (Qiagen, Hilden, Germany) at −80 °C until the pellets were processed and analyzed. Concentrations of ammonium sulfate in the supernatant were determined by a colorimetric method (Chaney and Marbach 1962)^35^. In summary, 1 mL of concentrated phenol–sodium nitroprusside solution and 1 mL of concentrated sodium hydroxide–sodium hypochlorite solution were added to the sample successively and mixed by vortexing. After incubating for 1 h at room temperature, samples were diluted and path–length corrected absorbances were measured at 625 nm with the BioTek Synergy 2 Multi–mode Microplate Reader. Utilization of peptides was measured indirectly by acid hydrolysis at high temperature in a N_2_ atmosphere (Griswold and Mackie 1997)^36^. The concentration of amino acids in the supernatants of tryptone–absent cultures and HCl–treated supernatants was then determined by the colorimetric ninhydrin method developed by Rosen^37^, using casamino acids as standard. Briefly, ammonium was removed by adding NaOH until pH > 10 and heating the samples until dry (approx. 1 h) in a vacuum concentrator (SpeedVac^TM^, Thermo Fisher, MA, USA). Samples were re–hydrated with 200 μL of water. Samples were treated with 100 μL CN–acetate buffer followed by 100 μL 3% ninhydrin in methyl cellosolve and heated at 100 °C for 15 min. Samples were immediately diluted with 50% isopropanol. Path–length corrected absorbances were measured at 570 nm and 440 nm with the BioTek Synergy 2 Multi–mode Plate Reader.

### Chemostat studies

Differences in the transcriptional profile driven by growth at different ammonium concentrations were evaluated in continuous culture in a Biostat B fermenter (B. Braun Biotech Inc., Allentown, PA) (Supplementary Fig. S2) by growth in excess [10 mM (NH_4_)_2_SO_4_] and limiting [0.7 mM (NH_4_)_2_SO_4_] ammonium conditions. The working volume was set at 1 L at 38.7 °C and pH 6.7. Agitation was provided by an impeller at 300 rpm, while anaerobic conditions were maintained with a continuous flow of O_2_–free CO_2_ through a sterile 0.22 µm gas filter. Growth was initiated by incubation as a batch culture for the first 12 hours with 10 mM (NH_4_)_2_SO_4_. After this time, continuous culture was started at a dilution rate of 0.17 h^–1^ ^9^. For RNA and protein extractions, cell cultures were harvested at mid–log phase by centrifugation at 10,000 x *g* for 5 min at 40, 70, 90 h for excess ammonium, and 130, 157, and 178 h for low ammonium. The medium reservoir was replaced with ammonium–limiting (0.7 mM) medium at 24 h intervals allowing a four volume turnover of medium between each sampling. The centrifuged cell pellet and supernatant were stored in RNAprotect bacteria reagent at –80 °C until the pellets were processed and analyzed.

### RNA extraction and purification

Total RNA was isolated from cells grown on different nitrogen sources and concentrations using Trizol (Invitrogen Corp., Carlsbad, CA). Briefly, the cell pellets were frozen under liquid N_2_ and ground with pre–chilled, sterile pestle and mortar. Trizol reagent was added to the grounded cell pellet and the mixture was homogenized by pipetting several times. Cellular proteins were further precipitated and lipids dissolved using chloroform and removed by centrifugation. Total RNA was then extracted with ethanol and sodium acetate, and resuspended with diethylpyrocarbonate (DEPC)–treated water. The RNA was further purified using a Qiagen RNeasy Cleanup kit (Qiagen, Valencia, CA) following the instructions provided by the manufacturer. RNA concentrations and quality were determined by measuring the absorbance ratio *A*_260_/*A*_280_ with Nanodrop (NanoDrop Technologies, Wilmington, DE) and using an Agilent 2100 bioanalyzer (Agilent Technologies, Palo Alto, CA) with RNA 6000 NANO assay. Only RNA with an *A*_260_/*A*_280_ ratio of >1.9 and RNA Integrity Number (RIN) >9.0 was used for cDNA synthesis.

### cDNA synthesis, labeling and hybridization in microarray

cDNA was synthesized from 0.1–10 μg of total RNA using random hexamers and the FairPlay III microarray labeling kit (Stratagene, La Jolla, CA) as per the manufacturer’s protocol. After cDNA synthesis, template RNA was removed and cDNA was purified using 4 μL of 3 M sodium acetate (pH 4.5), 1 μL 20 mg/ml glycogen and 100 μL 95% ethanol and incubated at −20 °C for 30 min. cDNA was centrifuged and washed with 0.5 mL of 70% ethanol and air–dried. Purified cDNA was labeled with either Cy3–dCTP or Cy5–dCTP dye (GE Healthcare, Piscataway, NJ) following the manufacturer’s protocol and purified by using the FairPlay III microarray labeling kits purification module. Purified cDNAs were hybridized to the custom Agilent 8 × 15 K microarrays as per the manufacturer’s protocol. Three biological replicates per microarray were analyzed, each with a dye swap. Each gene was present on the array 5 times, adding extra power to the statistical analysis. Microarray slides were scanned using a GenePix^®^ Professional 4200b scanner and GenePix Pro 6.0 software (Molecular Devices, Sunnyvale, CA, USA) and analyzed using the Limma package in Bioconductor^38^. Genes with an up– or down–regulation of 1.9 fold or greater and an FDR value < 0.05 were deemed to be statistically significant.

### Quantitative real–time PCR assay (qRT–PCR)

The cDNA was diluted 1:4 with DNase/RNase–free water. The RT–qPCR was performed using SYBR Green I (Applied Biosystems, Foster city, CA) with an ABI Prism 7900 High Throughput Sequence Detection System. Four microliters of cDNA was mixed with 5 μL SYBR Green master mix (Applied Biosystems), 0.4 μL of each forward and reverse primer (Supplementary Table S1), and 0.2 μL of DNase/RNase–free water. Each sample was run in triplicate. A standard curve of the internal control genes *atpD* (ORFB01230), *infB* (ORFB02450), and *rpoB* (ORFB02217) from cDNA microarray plus a non–template control (NTC) was used as reference. The RT–qPCR reactions were performed with the following conditions: 50 °C for 2 min, 95 °C for 10 min, and 40 cycles of 95 °C for 15 s and 60 °C for 1 min. In addition, to verify the presence of a single PCR product, a dissociation protocol using incremental temperatures to 95 °C for 15 s plus 65 °C for 15 s was performed. Data were analyzed using the SDS software version 2.2.1 (Applied Biosystems) using the six–point standard curve.

## Electronic supplementary material

Supplementary Information
