# Supplementary material for: Metabolic networks for nitrogen utilization in Prevotella ruminicola23
Source: Sci Rep. 2017 Aug 10;7:7851. doi: 10.1038/s41598-017-08463-3 (PMC5552732; doi:10.1038/s41598-017-08463-3)
Supplement: Supplementary file 1 — Supplementary Information [file 41598_2017_8463_MOESM1_ESM.pdf]

Supplementary Information

Metabolic networks for nitrogen utilization in *Prevotella ruminicola* 23

Jong Nam Kim[1], Celia Méndez–García[1], Renae R. Geier[1], Michael Iakiviak[1], Jongsoo Chang[2], Isaac Cann[1,3,4], Roderick I. Mackie[1,3].

[1]Department of Animal Sciences, University of Illinois, Urbana, Illinois, USA; [2]Department of Agricultural Sciences, Korean National Open University, Seoul, Korea; [3]Carl R. Woese Institute for Genomic Biology, University of Illinois, Urbana, Illinois, USA; [4]Department of Microbiology, University of Illinois, Urbana, USA.

Supplemental methods

Preparation of crude protein extract. Crude protein extracts were prepared using a modification of previously described methods [33,40]. Briefly, 1L of each culture sample (3 technical replicates per condition) was harvested by centrifugation at 10,000 × *g* for 20 min at 4 ºC. Pellets were washed once with an anaerobic buffer (50 mM Tris, 1% KCl, 1 mM Dithiothreitol, pH 6.8) [40], then resuspended in 10 mL of lysis buffer (20 mM Tris–HCl, 1 mM DTT, 1 mM phenylmethylsulfonyl fluoride) and subsequently disrupted by sonication at 80 W in 30 s cycles. Unbroken cells and cell debris were removed by centrifugation at 12,000 × *g* for 5 min at 4 ºC. Supernatant was transferred to new tubes and used for the separation of cytoplasmic and membrane–associated protein extracts. Cytoplasmic and membrane–associated protein extracts were prepared using a previously described method [41]. Crude protein extracts were subjected to ultracentrifugation in a fixed angle rotor at 105,000 × *g* for 90 min at 4 ºC. The supernatant following ultracentrifugation constituted the membrane–free cytoplasmic protein extract and the membrane–associated protein fraction was present in the pellet. The membrane associated fraction was resuspended with 3 mL of anaerobic buffer and then subjected to ultracentrifugation at 105,000 × g for 90 min at 4 ºC. The resulting pellet was resuspended to 1 mL of anaerobic buffer and this protein extract constituted the membrane protein extract.

2D–DIGE (Difference Gel Electrophoresis) and protein identification through mass spectrometry. Samples grown in ammonium or peptides were dissolved in standard cell lysis buffer containing 7 M urea, 2 M thiourea, 4% (w/v) CHAPs, and 30

mM Tris–HCl. Protein concentration was determined with 2–D Quant kit (GE Healthcare) according to the procedure described. For DIGE, the lysates were labeled with 400 pmol of Cy3 or Cy5 following the protocol described in the Ettan DIGE Manual (18–1164–40 Edition AA, GE Healthcare). For reproducibility, the pooled standard containing equal quantities of all the samples was labeled with 400 pmol of Cy2 in CyDIGE flour working solution. The internal control labeled with Cy2 and the protein samples labeled either with Cy3 or Cy5 were incubated for 30 min at 4 °C in CyDIGE flour working solution. After stopping the labeling reaction by adding 1 μL of 10 mM lysine to the mixture, the samples were incubated for 10 min at 4 °C in the dark. To achieve statistical confidence, protein labeling was repeated three times using dye swaps. The 2–D DIGE was performed using the IPGphor system as described in 2–D Electrophoresis manual (GE Healthcare). The IPG strips (24cm, pH3–10NL, GE Healthcare) were rehydrated and samples were labeled in the dark, overnight with rehydration buffer (8 M urea, 4% w/v CHAPS, 1% w/v pH 3–10 pharmalytes and 0.002% bromophenol blue). The first dimension was focused on an Ettan IPGphor system for a total of 88 kVh at 20 °C with a linear increase of voltage from 0 to 300 V, 600 V, 1000 V and 5000 V for 24 hr. The IPG strips were then equilibrated for 10 min in a buffer containing 50 mM Tris–HCl (pH 8.8), 6 M urea, 20% glycerol (w/v), 2% SDS (w/v), and 1% DTT (w/v). The strips were treated with the same solution containing 2.5% (w/v) iodoacetamide instead of DTT. The second dimension separation was performed on 12% SDS polyacrylamide gel upon application of 8 watts per gel using an Ettan DALT six system (GE Healthcare). For

image analysis, the Cy2, Cy3 and Cy5–labeled images were acquired on a Typhoon 9400 scanner (GE Healthcare) at the appreciate emission values of Cy2 (520nm), Cy3 (580nm) and Cy5 (670nm). The DIGE images were then analyzed using Decyder program V6.5 as described in the user manual (GE Healthcare). Intra–gel spot detection and quantification and inter–gel matching and quantification were performed using differential in–gel analysis (DIA) and biological variation analysis (BVA) modules, respectively. Only those spots with a > 3–fold change and t–test <0.05 among treatments were picked and analyzed by LC–MS/MS for protein identification. The excised protein spots were digested in–gel with trypsin (Promega, Madison, WI). Briefly, gel pieces were destained in 50% methanol and 5% acetic acid and then treated using an alkylation and reduction process with 10 mM of DTT and 55 mM of iodoacetic acid, respectively. Following vacuum drying, the gel pieces were pre–incubated in 20 µg/mL trypsin solution for 45 min at 4 ºC, then incubated in 50 mM NH_4HCO_3 overnight at 37ºC. An extraction buffer (5% TFA in 50% acetonitrile) was added to supernatants and the pellet was collected by centrifugation and subsequently vacuum–dried. The peptides were concentrated with Zip tip µ–c18 pipette tips (Millipore, Bedford, MA). The enzymatically digested samples were then analyzed by a hybrid Quadrupole–TOF MS/MS spectrometer (Applied Biosystems). Peptides were separated on a Zorbax 300SB–C18 capillary column (Agilent) at a flow rate of 300 nL/min. The resulting peptides were electro–sprayed through a coated silica tip (New Objective, Woburn, MA) at an ion spray voltage of 2,300 eV. The mass data were acquired automatically using Analyst QS

2.0 software (Applied Biosystems). For data acquisition, the mass spectrometer was set in the positive ion mode at a selected mass range of 400–1600 m/z for a 1 sec TOF–MS survey scan to detect precursor ions.

Enzyme activity assay. Enzyme activities of NADH– and NADPH–dependent glutamate dehydrogenase (GDH), biosynthetic glutamine synthetase (GS), NADH– and NADPH–dependent glutamate synthase (GOGAT) were measured using previously described methods [5,7-11]. The assay mixture for the biosynthetic reaction measuring the ability of the GS to form glutamine by detection of the P_i released from ATP consisted of 100 mM MOPS (pH 7.5), 50 mM $MgCl_2 \cdot 6H_2O$, 250 mM L–glutamate, 50 mM NH_4Cl to which approximately 10 μg of GSI, GSIII–1, or GSIII–2 was added. The total 90 µL of mixture was equilibrated at 37°C for 5 min, and the reaction was initiated by adding 10 µL of 0.1 M ATP (final concentration of 10 mM) in a total volume of 100 µL. Twenty-five microliters of the reaction mixture was transferred after 5 min to a microtiter plate and 75 µL of solution D (mixture of two parts of 12% (w/v) L–ascorbic acids in 1N HCl and one part 2% (w/v) ammonium molybdate tetrahydrate in ddH_2O) was added. After 5 min of incubation, 75 µL of stop color development solution F (2% (w/v) sodium citrate tribasic dehydrate, 2% (v/v) acetic acid, and 2% (w/v) sodium meta–arsenite, in ddH_2O) was added and the solution was incubated for 15 min at 37 ºC. A blank was prepared using 10 µL of ddH_2O and 90 µL of enzyme mixture. The inorganic phosphate product was measured spectrophotometrically at 850 nm as for the biosynthetic assay. GS specific activity is expressed as nmolP_i/µg/min

Supplemental Tables

SI Table 1. List of primers used for qRT-PCR.

Primers	Annotation	Sequence (5' — 3')
amt-F	Ammonium transporter	GGCAAGTATGATGAGGACGGTAA
amt-R		ATCCGAACCATCCCAACCA
glnK-F	nitrogen regulatory protein P_{II}	GTGTGGCCATCACCATTGTG
glnK-R		ATACGACCGTCGCCGATTT
nextamt-F	conserved hypothetical protein	CAACATCGGCTTGACCGACTA
nextamt-R		TGCCTCGAAGATATGGTTGGT
gltB-F	NADPH-GOGAT, large subunit	AAAGAAAGCAAACTCAACCCAAA
gltB-R		CTTTTTCCGCCGTGTATGTTAAC
gltD-F	NADH-NADPH GOGAT, small subunit	AGGACGAGGCTGCCATCAC
gltD-R		GCACCAATCACAGCCACCTT
gdhA-F	NADPH-GDH	ATGCTGGCTACTCGCGGTAT
gdhA-R		CAAGCTGCAGGCACTTCTCA
gdh-F	NADH-GDH	CACTTACCACCCTGCCTATGG
gdh-R		ATGAATGCCTGGCAGAAACG
GSI-F	Glutamine synthetase I	AGGCGCCCACTAATGTTTGT
GSI-R		TTCGCAGCATGACACATATCC
glnA-F	Glutamine synthetase III-1	CGGATGGGAACAGGAGTACTTC
glnA-R		GGCACTATCGTGTCCCATCAG
glnN-F	Glutamine synthetase III-2	CACATCGCCATTTGCCTTTA
glnN-R		CACAGCCGAGTTAAGTGCAATC
dapF-F	diaminopimelate epimerase	GGCAATCCCCACTACGTGAT
dapF-R		TGTTACATCTTTGTGGGAATGCA
asnB-F	asparagine synthase	AGCCGACGAATTCCTGATTG
asnB-R		TTTCAGCTCCGAGGCTACGT
amitrans-F	aminotransferase	GCTGCCGAGGCCATCTATAC
amitrans-R		CACGCAGGGTGGTAAGCAT
diamino-F	diaminopimelate dehydrogenase	ATGTCGATGGGCCACAGTGT
diamino-R		CCATACGGCGGTGGATACC
ragA-F	receptor antigen RagA	GGGCTGGTTCCCATCACTT
ragA-R		AATGCTGGCACGCAGTTTC
acser-F	O-acetylhomoserine aminocarboxypropyltransferase	GGACTTCGCGATGCTGGTAA
acser-R		GACCTGCTACTCCACCTTCGA
cysK-F	cysteine synthase A	GCACTGGTATCAGCCGTCAA
cysK-R		CCTCGGCACCATAGGCTTT
luxR-F	transcriptional regulator, LuxR family	ACCAAGGAGATTGCCGAAGA
luxR-R		GTTCGTGGGCTGTGTTGATG
rplR-F	ribosomal protein L18	GGGTCTCGAGGCTATGCCTAA
rplR-R		GTCGAAGACAACGGCGCTAA
rpsK-F	ribosomal protein S11	TGCGCTAAGGTTGCTTACGA
rpsK-R		CACCATGAATGGCACGGATA
infA-F	translation initiation factor IF-1	TGAGCAGGACGGAACAATTGT
infA-R		CATCTTACCAGAGATATGCGCAATA
rpmD-F	ribosomal protein L30	CAGTTGATCAGAAGCGCACTCT
rpmD-R		TTACGGATCATACCACGGATTG
atpD-F	ATP synthase F1, beta subunit	TGGGTATCTATCCCGCTGTTG
atpD-R		TTGACACGCTGGGCACAAT

infB-F	translation initiation factor IF-2	TCAGAGCTGGCCACCATGA
infB-R		CAGCATCCAGACGCTGGTT
rpoB-F	DNA-directed RNA polymerase, beta subunit	GAAGACCTTGCTGAGTGGACTGA
rpoB-R		TAGCAGGCTGGTCGAAACG

SI Table 2. Differentially expressed genes in *P. ruminicola* 23 grown from non-limiting to limiting ammonium concentrations in chemostat culture.

ORF number	Gene or locus tag	Gene function	Primary category role*	EC number	Fold change	
					Microarray	qRT-PCR
ORFB02055	*amt*	ammonium transporter	TBP		69.1	387.1
ORFB02056	PRU_1977	conserved hypothetical protein	HP		58.3	1130.3
ORFB02054	*glnK*	nitrogen regulatory protein P-II	RF		46.2	281.0
ORFB02058	*asnB*	asparagine synthase (glutamine-hydrolyzing)	AAB	6.3.5.4	38.0	159.3
ORFB02034	*glnA*	glutamine synthetase, type III [GSIII-2]	AAB	6.3.1.2	35.4	71.3
ORFB02035	*dapF*	diaminopimelate epimerase	AAB	5.1.1.7	33.1	73.5
ORFB02037	*gltD*	glutamate synthase, NADH/NADPH, small subunit	AAB	1.4.1.-	32.6	124.0
ORFB02039	*gltA*	glutamate synthase (NADPH), large subunit	AAB	1.4.1.13	26.6	17.0
ORFB01303		lipoprotein, putative	CE		10.3	
ORFB02042		antirepressor, putative	RF		10.2	
ORFB01305		TonB dependent receptor	TBP		9.2	
ORFB02053	PRU_1974	aminotransferase, homolog	UF		7.8	13.5
ORFB02902		conserved domain protein	HP		7.0	
ORFB02903		lipoprotein, putative	CE		6.3	
ORFB02040		Hypothetical protein	HP		6.3	
ORFB01729		conserved hypothetical protein	HP		5.1	
ORFB02052		glutamine amidotransferase, class-II domain protein	UF		5.0	3.7
ORFB01956		Hypothetical protein	HP		5.0	
ORFB02002		outer membrane efflux protein	TBP		4.8	
ORFB02438		Hypothetical protein	HP		4.8	
ORFB02011		membrane protein, putative	CE		4.7	
ORFB02041		Hypothetical protein	HP		4.5	
ORFB02004		efflux transporter, RND family, MFP subunit	TBP		4.4	
ORFB02439		CUB domain protein	UF		4.4	
ORFB01408		Hypothetical protein	HP		4.3	
ORFB02010		membrane protein, putative	CE		4.2	
ORFB01407	*nrdG*	anaerobic ribonucleoside-triphosphate reductase activating protein	PPNN		4.1	

ORFB02008		ABC transporter, ATP-binding protein	TBP		4.1	
ORFB02005		ABC transporter, permease protein	TBP		4.1	
ORFB02256		alpha-1,2-mannosidase family protein	CE		4.0	
ORFB01152		lipoprotein, putative	CE		-4.0	
ORFB02397		Hypothetical protein	HP		-4.0	
ORFB02354		sugar transporter, fucose:hydrogen symporter (FHS) family	TBP		-4.0	
ORFB01800		conserved hypothetical protein	HP		-4.1	
ORFB01114		transporter, outer membrane receptor (OMR) family	TBP		-4.1	
ORFB02355		fructokinase, putative	EM		-4.2	
ORFB00980		Hypothetical protein	HP		-4.2	
ORFB01642		receptor antigen RagA, putative	CE		-4.2	
ORFB01151		membrane protein, putative	CE		-4.3	
ORFB00996		Hypothetical protein	HP		-4.5	
ORFB00670		Hypothetical protein	HP		-4.5	
ORFB02926		transcriptional regulator, LuxR family	RF		-4.6	-15.5
ORFB00650		Hypothetical protein	HP		-4.8	
ORFB00651		conserved hypothetical protein	HP		-5.1	
ORFB00916	*ssb*	single-strand binding protein	DM		-5.3	
ORFB01380		conserved hypothetical protein	HP		-5.4	
ORFB02352		glycosyl hydrolase, family 32	EM		-5.5	
ORFB02935		transporter, outer membrane receptor (OMR) family	TBP		-5.6	
ORFB00915		gliding motility protein GldE	CP		-5.8	
ORFB02349		conserved hypothetical protein	HP		-5.9	
ORFB02387		Hypothetical protein	HP		-6.0	
ORFB00917		conserved domain protein	HP		-6.3	
ORFB02351		lipoprotein, putative	CE		-7.0	
ORFB00667		RNA polymerase sigma-70 factor family protein	TR		-7.5	
ORFB02350		lipoprotein, putative	CE		-8.0	
ORFB02894	*cysK*	cysteine synthase A	AAB	2.5.1.47	-10.1	-17.5
ORFB00666		Hypothetical protein	HP		-10.2	
ORFB02893	PRU_2791	O-acetylhomoserine aminocarboxypropyltransferase	AAB		-13.2	-31.4
ORFB02928		Hypothetical protein	HP		-13.9	
ORFB02934		lipoprotein, putative	CE		-14.7	
ORFB02933		conserved domain protein	HP		-16.4	
ORFB02930		lipoprotein, putative	CE		-17.1	

ORFB02931		Hypothetical protein	HP		-18.6	
ORFB02929	PRU_2827	receptor antigen RagA, putative	CE		-18.7	-51.5
ORFB02932		Hypothetical protein	HP		-19.1	

* Primary categories legend: AAB, Amino acid biosynthesis; BCPC, Biosynthesis of cofactors, prosthetic groups, and carriers; CE, Cell envelope; CP, Cellular processes; CIM, Central intermediary metabolism; DM, DNA metabolism; EM, Energy Metabolism; FP, Fatty acid and phospholipid metabolism; MEEF, Mobile and extrachromosomal element functions; PF, Protein fate; PS, Protein synthesis; PPNN, Purines, pyrimidines, nucleosides, and nucleotides; RF, Regulatory functions; ST, Signal transduction; TR, Transcription; TBP, Transport and binding proteins; UF, Unknown function.

SI Table 3. Differentially expressed genes in *P. ruminicola* 23 grown on different nitrogen sources. Fold changes correspond to growth on ammonium as compared to growth on peptides

ORF number	Gene or locus tag	Gene function	Primary category role*	EC number	Fold change	
					Microarray	qRT-PCR
ORFB02055	*amt*	ammonium transporter	TBP		47.0	96.1
ORFB02056	PRU_1977	conserved hypothetical protein	CHP		46.5	243.6
ORFB02054	*glnK*	nitrogen regulatory protein P_{II}	RF		43.3	145.2
ORFB02039	*gltA*	glutamate synthase (NADPH), large subunit	AAB	1.4.1.13	26.3	32.9
ORFB02035	*dapF*	diaminopimelate epimerase	AAB	5.1.1.7	22.7	26.0
ORFB02034	*glnA*	glutamine synthetase, type III	AAB	6.3.1.2	22.5	105.4
ORFB02037	*gltD*	glutamate synthase, NADH/NADPH, small subunit	AAB	1.4.1.-	22.4	39.3
ORFB02058	*asnB*	asparagine synthase (glutamine-hydrolyzing)	AAB	6.3.5.4	15.3	63.1
ORFB02053	PRU_1974	aminotransferase, homolog	UF		13.0	18.5
ORFB02120	PRU_2042	diaminopimelate dehydrogenase	AAB	1.4.1.16	9.9	17.7
ORFB02051	*pyrG*	CTP synthase	PPNN	6.3.4.2	6.9	
ORFB02052	PRU_1973	glutamine amidotransferase, class-II domain protein	UF		6.8	9.0
ORFB02864	PRU_2766	phosphomethylpyrimidine kinase, putative/transcriptional regulator, AraC family	BCPC		6.3	
ORFB01958		lipoprotein, putative	CE		5.7	
ORFB01957		conserved hypothetical protein	CHP		5.3	
ORFB02121	*purN*	phosphoribosylglycinamide formyltransferase	PPNN	2.1.2.2	4.6	8.0
ORFB02866	PRU_2767	pyridoxine biosynthesis protein	BCPC		4.6	
ORFB02867		glutamine amidotransferase, SNO family	UF		4.4	
ORFB02926		transcriptional regulator, LuxR family	RF		-4.7	-3.1
ORFB02896		hypothetical protein	HP		-4.8	
ORFB02928		hypothetical protein	HP		-5.5	
ORFB02930		lipoprotein, putatuve	CE		-6.2	
ORFB02893	PRU_2791	O-acetylhomoserine aminocarboxypropyltransferase/cysteine synthase family protein	AAB		-7.5	-138.2
ORFB02931		hypothetical protein	HP		-7.6	
ORFB02934		lipoprotein, putative	CE		-7.8	
ORFB02932		hypothetical protein	HP		-7.8	
ORFB02929	PRU_2827	receptor antigen RagA, putative	CE		-8.0	-11.4

ORFB02933		conserved domain protein	CHP		-9.0	
ORFB02894	*cysK*	cysteine synthase A	AAB	2.5.1.47	-12.0	-83.5

* Primary categories legend: AAB, Amino acid biosynthesis; BCPC, Biosynthesis of cofactors, prosthetic groups, and carriers; CE, Cell envelope; CP, Cellular processes; CIM, Central intermediary metabolism; DM, DNA metabolism; EM, Energy Metabolism; FP, Fatty acid and phospholipid metabolism; MEEF, Mobile and extrachromosomal element functions; PF, Protein fate; PS, Protein synthesis; PPNN, Purines, pyrimidines, nucleosides, and nucleotides; RF, Regulatory functions; ST, Signal transduction; TR, Transcription; TBP, Transport and binding proteins; UF, Unknown function.

SI Table 4. List of identified cytoplasmic proteins differentially expressed in *P. ruminicola* 23 on ammonia and peptides.

Spot ID	Treatment	Fold change [a]	t-test	ANOVA	ORF number	Annotation
456	Peptides	68.9	0.001	0.001	ORFB02894	Cysteine synthetase A (*cysK*)
595	Peptides	8.5	0.009	0.009	ORFB02893	O-acetylhomoserine aminocarboxypropyltransferase
598	Peptides	24.5	0.004	0.004		
104	Ammonia	13.3	0.001	0.001		
108	Ammonia	29.1	0.001	0.001		
111	Ammonia	57.2	0.001	0.001		
112	Ammonia	37.8	0.001	0.001	ORFB02034	Glutamine synthetase, Type III (GSIII-2)
212	Ammonia	11.9	0.02	0.02		
329	Ammonia	4.2	0.009	0.009	ORFB02037	Glutamate synthase
462	Ammonia	5.2	0.02	0.02		

Supplemental Figures

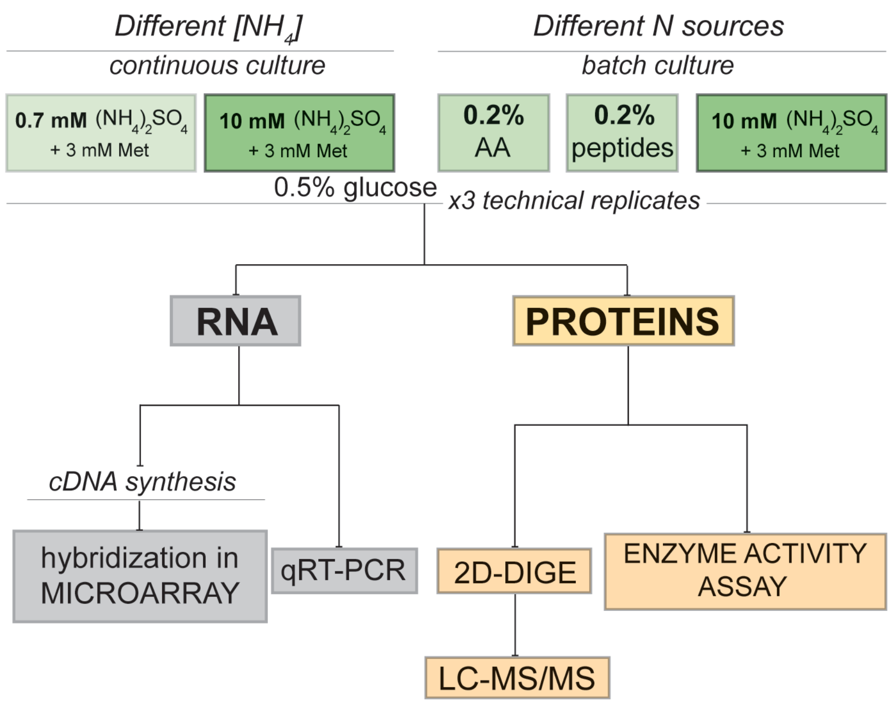

Figure S1. Schematic of the experimental design followed for the determination of transcriptional responses to environmental nitrogen changes in *P. ruminicola* 23. Abbreviations: AA, Amino Acids; 2D-DIGE, 2D Difference Gel Electrophoresis.

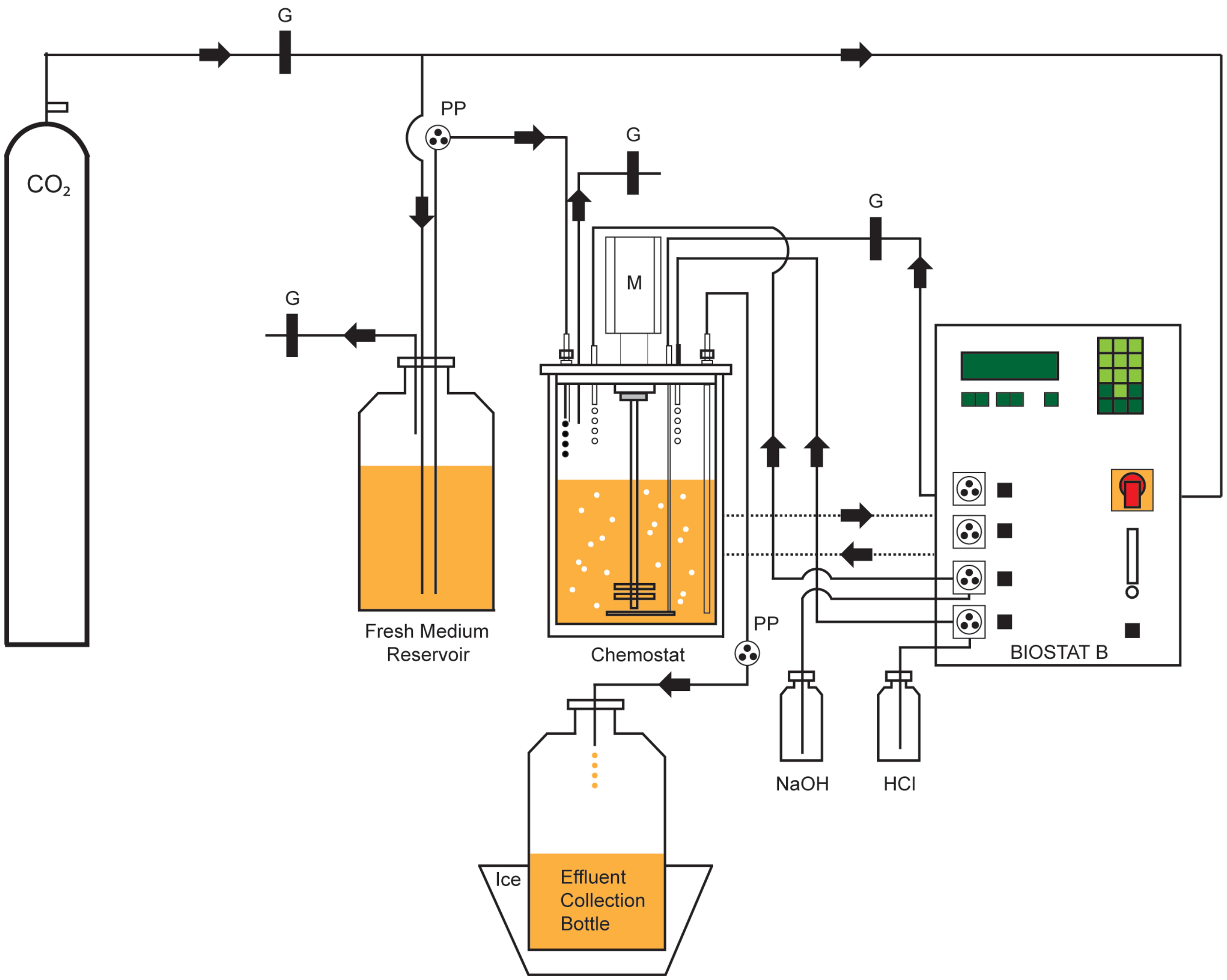

Figure S2. Schematic diagram of the continuous culture system: G, Sterile air filter; PP, peristaltic pump; M, overhead impeller drive motor; solid lines, gas flow and medium flow; dotted lines, temperature controller. Arrows show direction of medium or gas flow.

PRU_1977

```
              o > i
MKKIVLMALMFAAGMGASAQDEVERLRVGERSSGMTTVAADVVSSYIWRGQDCGSAAIQPTLGIGYKGLS
              i > o
LTAWGSYGLVDTNDAKEFDLTLAYTAGGFNIGLTDYWFNAGLDPEGRYFKYDAHGTNHIFEANIGYDFGAA
                           o > i                       i > o
SIQWFTNLSGNDGVNKDGKRAYSSYVELGVPFKVAAVDWSATVGAVPYATSFYGTDGFAITNLALKATKDI
                                                       i > o
KVTDSFSIPVFAQVAANPCAQKAYFVFGFTLQP
```

Figure S3. Predictive transmembrane helices for the hypothetical conserved protein PRU_1977, located immediately downstream of the ammonium transporter gene, *amtB* and highly induced in non-limiting ammonium concentrations. Underlined in green, most probable helices. Yellow lines represent the second most probable set of transmembrane domains. “i > o” and “o > i” represent the orientation of the domain (inside towards outside of the cell membrane and outside to inside, respectively).

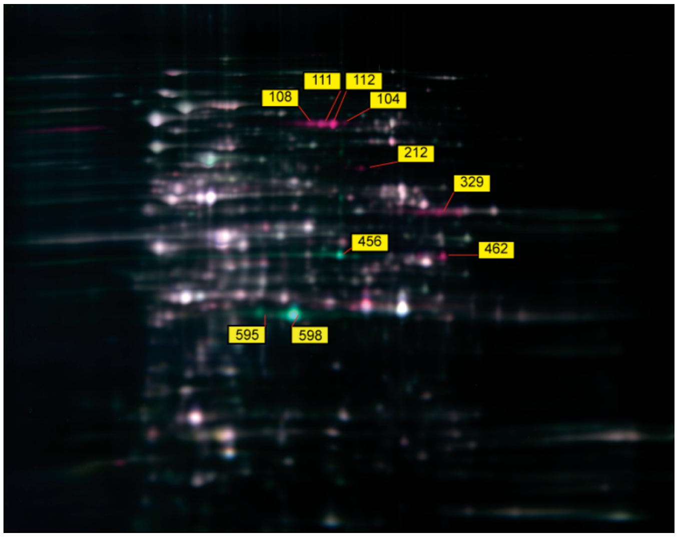

Figure S4. Overlay image of two-dimensional difference gel electrophoresis (2D-DIGE) of cytoplasmic proteins in *P. ruminicola* 23 grown on ammonia (red spots – numbers 104, 108, 111, 112, 212, 329, 462) or peptides (blue spots – numbers 456, 595, 598).
